# Supplementary material for: Bundle recommendation methods considering rating data differences for online retailers
Source: PLoS One. 2025 Sep 3;20(9):e0328245. doi: 10.1371/journal.pone.0328245 (PMC12407470; doi:10.1371/journal.pone.0328245)
Supplement: Attachment — Submitted filename: response.docx [file pone.0328245.s003.docx]

***Note to Reviewers*:** We sincerely appreciate the time and effort you put into reviewing our paper and for your detailed and constructive comments. We have revised the manuscript according to your suggestions. The following pages describe how we have responded to each of your comments. Note that your comments are written in *blue italics*, whereas our responses are given in regular black fonts.

*Reviewer #1: There are several things that I recommend the authors to check:

(1) Formula (4), as I understand from the formula, the range is no longer between 0 to 1 as in the initial cosine similarity. In my understanding, rev1 and rev2 can be considered as weights. rev_1 is between 0 and 1, so it seems OK, but rev_2 is between e and 1; as these two are the multiplier of the initial cosine similarity, then the similarity is no longer between 0 and 1. Moreover, if the item is popular, then Ni is large, then rev2 will to 1; but if the item is not popular then rev2 will close e, which will increase the similarity, i.e increase the sim (u.v), according to Formula (4). Isn't this contradictory?*

Thank you for your carefully checking and kind review. We sincerely apologize for the typographical error in Equations (3) which brings you confusions. The calculation of *rev2* should be ${rev}_{2}=e^{\frac{N(i)}{\left| U \right|}-1}$, instead of $e^{1-\frac{N(i)}{\left| U \right|}}$. Then it ensures that the resulting coefficient in Equation (4) strictly adheres to the [0,1] interval, consistent with the theoretical design.

We have revised Equation (3) in the manuscript and highlighted the revisions. Please refer to **Section 4.2.3 (Page 15)** to see the details.

*(2) It is stated following Formula (4) that the RHS is the classic cosine similarity. Well, I disagree with this, since it's the modified, since it is already weighed by rev1 and rev2*

Thank you for your attentive review of the formula description. The ambiguity caused by the original statement regarding "the classic cosine similarity calculation" has led to a misinterpretation of the equation's structure. We have revised the description as *"**The rightmost fractional component in the equation corresponds to the standard cosine similarity calculation."*

This revision does not alter the validity of subsequent analyses, and the revision is highlighted on **Section 4.2.3 (Page 15)** in the revised manuscript. You may kindly check the details.

*(3) Formula (5): Ni(u) is the neighbors set of rating the same items with user u; does it mean they rate all the same items? Or it can be just some items are the same, and some others can be different? Or just the specified item-i? What if Ni(u) is an empty set? Moreover, please check the consequence of my concern about Formula (4) on the similarity in Formula (5).*

Your profound analysis of the formula's applicability boundaries has significantly advanced this research. Equation (5) calculates the predicted rating ${\hat{\boldsymbol{r}}}_{u,i}^{cf}$ for user $u$ to item $i$.

$${\hat{\boldsymbol{r}}}_{u,i}^{cf}=\frac{\sum_{v\in N_{i\left( u \right)}} sim\left( \vec{u}\cdot\vec{v} \right)r_{v,i}}{\sum_{v\in N_{i\left( u \right)}} sim\left( \vec{u}\cdot\vec{v} \right)}$$

(5)

$N_{i\left( u \right)}$ is the top-N nearest neighbor user set to user $u$. $r_{v,i}$ is the actual rating score of user $v$ to item $i$, where $v\in N_{i\left( u \right)}$ and $sim\left( \vec{u}\cdot\vec{v} \right)$ is the similarity between user $u$ and $v$. It does not require that these users have rated all the same items. In order to prevent the neighbor set *Ni(u)* from being empty, the data of users with extremely few review records and items with few ratings has to be deleted. Then, appropriately select the neighborhood size is essential. Details regarding size configuration are addressed in Question 9. We have made detailed explanations. Please kindly refer to **Section 4.2.3 (Page 15) and Section 5.1 (Page 25)**.

*(4) Page 14: dimension K --> what is K? how to determine K? is it related to the index k?*

Your critical insights regarding both the definition and selection method of this parameter have been indispensable to our research.

Here, $\boldsymbol{K}$ represents the dimensionality of the latent factor space to which users and items are mapped. To avoid confusion with a specific feature dimension $k$ (e.g., the *k*-th feature dimension), we consistently adopt $\boldsymbol{F}$ instead of $\boldsymbol{K}$ to denote the dimensionality of the latent space starting from **Section 4.2.3 (Page 16)**.

To ensure effective dimensionality reduction and model performance, the value of $\boldsymbol{F}$ must be carefully tuned through parametric analysis. In **Section 5.2.1 (Page 26)**, we defined a candidate set {5, 15, 25, 35, 45} based on historical experimental precedents, and subsequently validated *F=*35 as the optimal choice through hyperparameter analysis in **Section 5.2.4 (Page 27)**. You may check the details in the revised manuscript.

*(5) u=average rating --> how do you get the value? averaged over what?*

Your kindly and carefully review is appreciated. As for Equation (7), $\mu$ is the average rating of all items on the shopping platform or the dataset. For example, the average rating of all items on the dataset of Amazon.com. It is used to correct the systematic bias for different platforms or datasets.

$$\hat{r}_{u,i}^{svd++}=q_{i}^{T}\cdot\left( p_{u}+\frac{1}{\sqrt{N\left( u \right)}}\sum_{j\in N\left( u \right)} y_{j} \right)+bu+bi+\mu$$

(7)

We have provided an extended elaboration of this parameter to facilitate enhanced comprehension. Hope this time, it can be better understood. Would you kindly refer to **Section 4.2.3 (Page 17)** to see the details.

*(6) |Test| --> what is this? An explanation is required.*

We are humbled by your fastidious attention on explanations we had previously overlooked.

In Equation (10) and (11), the “$Test$” is the test set, and $|Test|$ indicates the number of *user-item* pairs in the test set. Here the dataset was randomly divided, with 20% allocated to the test set and 80% to the training set. The details explanations have been marked in **Section 4.2.3 (Page 18)**.

$$RMSE=\sqrt{\frac{\sum_{u,i\in Test} \left( \hat{r}_{u,i}-r_{u,i} \right)^{2}}{\left| Test \right|}}$$

(10)

$$MAE=\frac{\sum_{u,i\in Test} \left| \hat{r}_{u,i}-r_{u,i} \right|}{\left| Test \right|}$$

(11)

*(7) Page22: Formula (16) loss function: y.ycap --> is it dot product? isn't y the rating, i.e scalar? Or is it a vector? Is it related to y_j= the feature items that user has purchased but not rated? If there is no value yet to y_j, then how can we do y.yhat?*

Your insightful suggestions and critical questions have significantly contributed to the improvement of this manuscript.

Regarding the term in the loss function, the expression $y\cdot\hat{y}$ denotes the dot product between the true label vector $y$ and predicted label vector ŷ, which evaluates the classification task performance in the recommendation system. The hinge loss function here are independent of $y_{j}$ in the rating prediction module, where $y_{j}$ specifically participates in rating matrix completion.

In order to prevent ambiguity, we have made some corrections on the variable representations as follows. where$a$denotes the true label, and $\hat{a}$ denotes predicted label vector. You may refer to **Section 4.3.2 (Page 23)** to check the details.

$$loss\left( a \right)=\max\left( 0,1-a\cdot\hat{a} \right)$$

(16)

*(8) Page 23. Formula (17) for sparsity, the part "(number of users) x (number of items)" --> is it standard formula? Otherwise, why is it not just 1- #rated items/#items? Since if using that formula, even if all the items are rated, there is still sparsity, that is 1-1/#users --> this is kind of not really representing the idea of sparsity. Or is there other interpretation, or rationale of the sparsity in this context?*

We sincerely appreciate your insightful interpretation and suggestions on the formulation. Formula (17) is widely used in collaborative filtering and recommender system [1]. This formula reflects how full the user–item rating matrix is compared to its maximum possible size. In an ideal situation, each user rates each item, the matrix will be filled and the *Sparsity* will be 0. In fact, only a small part of items is actually rated. Therefore, we measure the sparsity degree through Equation (17). According to your advice, we have made some corrections on the variable representations and explanations as follows,

$$Sparsity=1-\frac{N_{ratings}}{|U|\times|I|}$$

(17)

where $N_{ratings}$ denotes the number of ratings, while ∣U∣ and ∣I∣ represent the total number of users and items respectively. Details can be accessed in **Section 5.1(Page 25)**.

[1] Idrissi N, Zellou A. A systematic literature review of sparsity issues in recommender systems. Soc. Netw. Anal. Min. 10, 15 (2020). https://doi.org/10.1007/s13278-020-0626-2.

*(9) The number of nearest neighbors for users was set to 50 --> the rationale? as in kNN usually we use 5; but there might be specific consideration in this context of data? The concern is that the larger the number of neighbors used, the more heterogeneous the result will likely.*

We sincerely appreciate your expert advice from a technical perspective. We set the number of nearest neighbors for users as 50. Because the user-item interaction data in our study is relatively sparse, and individual users often have limited interaction records. Using only a small number of neighbors may leads to unstable representations, especially in sparse settings. Also a trade-off between overfitting and underfitting has to be considered. Consequently, a relative larger number of neighbors is needed. This design choice is also supported by previous works that employ relatively large neighborhood sizes to combat sparsity and enhance model generalization [2,3].

According to your advice, we have explained it in the revised manuscript. Please refer to **Section 5.2.1 (Page 26)** to see the details.

[2] Koren Y. Factorization meets the neighborhood: a multifaceted collaborative filtering model. In Proceedings of the ACM SIGKDD International Conference on Knowledge Discovery and Data Mining. ACM, Las Vegas, Nevada, USA, 24-27 Aug. 2008.

[3] Sun F, Liu J, Wu J, Pei C, Lin X, Ou W, Jiang P. BERT4Rec: Sequential Recommendation with Bidirectional Encoder Representations from Transformer. arXiv:1904.06690,2019.

*(10) Page28: Formula (18), average of average? I think the formula is wrong, the 1st average is not needed.*

Thank you for highlighting this concern. Upon re-examination, we acknowledge that the original equation lacked precision in its formulation. We have refined the mathematical expression and added explicit definitions for all symbols involved. Here $\bar{r}_{it}$ represents the average rating of recommended items in bundle $t$, $\bar{r_{t}}$ represents the average rating of all recommended bundles, and $\bar{r_{u}}$ represents the historical average rating of users.

$$Satisfcation level=\frac{1}{n}\sum_{u=1}^{n} \frac{\bar{r_{t}}}{\bar{r_{u}}}$$

(18)

$$\bar{r_{t}}=\frac{1}{m}\sum_{t=1}^{m} \bar{r}_{it} , i\in t$$

(19)

We have made the corrections in the manuscript. Would you kindly check them in **Section 5.3.3 (Page 31).**

In addition to addressing the aforementioned issues, we have meticulously refined the manuscript's language. We once again express our deepest gratitude to the reviewers for your invaluable insights and suggestions.
